# Quartz Enhanced Photoacoustic Spectroscopy Based Trace Gas Sensors Using Different Quartz Tuning Forks

**DOI:** 10.3390/s150407596

**Published:** 2015-03-27

**Authors:** Yufei Ma, Guang Yu, Jingbo Zhang, Xin Yu, Rui Sun, Frank K. Tittel

**Affiliations:** 1 National Key Laboratory of Science and Technology on Tunable Laser, Harbin Institute of Technology, Harbin 150001, China; E-Mails: yuguang@hit.edu.cn (G.Y.); wozhenkule@sina.com (J.Z.); 2 Post-doctoral Mobile Station of Power Engineering and Engineering Thermophysics, Harbin Institute of Technology, Harbin 150001, China; E-Mail: sunsr@hit.edu.cn; 3 Department of Electrical and Computer Engineering, Rice University, 6100 Main Street, Houston, TX 77005, USA; E-Mail: fkt@rice.edu

**Keywords:** QEPAS, quartz tuning fork, resonant frequency, H_2_O quantification

## Abstract

A sensitive trace gas sensor platform based on quartz-enhanced photoacoustic spectroscopy (QEPAS) is reported. A 1.395 μm continuous wave (CW), distributed feedback pigtailed diode laser was used as the excitation source and H_2_O was selected as the target analyte. Two kinds of quartz tuning forks (QTFs) with a resonant frequency (*f_0_*) of 30.72 kHz and 38 kHz were employed for the first time as an acoustic wave transducer, respectively for QEPAS instead of a standard QTF with a *f_0_* of 32.768 kHz. The QEPAS sensor performance using the three different QTFs was experimentally investigated and theoretically analyzed. A minimum detection limit of 5.9 ppmv and 4.3 ppmv was achieved for *f_0_* of 32.768 kHz and 30.72 kHz, respectively.

## 1. Introduction

Trace gas sensor technologies are widely used in many applications, such as atmospheric chemistry [1,2], life science [3], medical diagnostics [4] and planetary exploration [5]. Trace gas sensor platforms based on near-infrared laser absorption spectroscopy have been reported in recent years [6,7,8,9,10]. Among these methods, photoacoustic spectroscopy (PAS) is an effective trace gas sensor technology which employs a broadband microphone for acoustic wave detection. For example, when the output of a near-infrared semiconductor laser is absorbed by a gas sample, the absorbed energy is transformed to heat energy by non-radiative processes, and will result in an increase of the local temperature and pressure in the sample. Therefore the absorption of a modulated near-infrared laser beam in a gas sample leads to the generation of an acoustic wave. The intensity of the acoustic wave is related to the sample concentration which can be detected by a sensitive microphone. However, most microphone-based PAS cells have a low resonance frequency, which makes cells more sensitive to environmental and sample gas flow noise. Moreover, the size of the typical photoacoustic cell is also relatively large [11].

A recent improvement of microphone-based PAS is quartz-enhanced photoacoustic spectroscopy (QEPAS) technique, which was first reported in 2002 [12]. This technique uses a low cost, commercially available mm sized piezoelectric quartz tuning fork (QTF) as an acoustic wave transducer which possesses a high detection sensitivity and immunity to ambient acoustic noise [13]. In QEPAS technology, the acoustic energy is accumulated in the sharply resonant QTF, and not in a larger photoacoustic cell as in conventional PAS. Therefore, a size limitation of the gas cell no longer exists and the cell volume can be reduced significantly, and even the gas cell can be optional. The total volume of a typical QEPAS acoustic detection module (ADM) is ~4 cm^3^. However, the ADM can be further reduced to ~3 mm^3^, because the volume of the analyzed gas sample is only limited by the dimensions of the QTF and the acoustic micro-resonator (mR) tubes. QEPAS has been successfully applied to trace gases detection in numerous applications [14,15,16,17,18,19,20], due to its advantages of high sensitivity, selectivity and compactness. The primary noise source in QEPAS is the thermal noise associated with the QTF at the resonant frequency *f_0_*. The QTF thermal noise can be expressed in terms of its root mean square (rms) voltage noise [21]:

(1) $$\sqrt{V_{rms}^{2}}=R_{g}\sqrt{\frac{4k_{B}T}{R}}\sqrt{\text{Δ}f}$$

(2) $$R=\frac{1}{Q}\sqrt{\frac{L}{C}}$$

where *k_B_* is the Boltzmann constant, *T* is QTF temperature, *R_g_* is the value of the feedback resistor used in a transimpedance amplifier located close to the ADM, Δ*f* is the detection bandwidth, and *R*, *L*, and *C* are the equivalent electrical parameters of resistance, inductance, and capacitance respectively for the QTF when it is represented by the equivalent serial resonant circuit. From Equations (1) and (2), we can determine the QEPAS noise level from the QTF parameters. The QEPAS thermal noise is reduced with increasing of *R*.

The QTF possesses a large dynamic range of nine orders of magnitude of the acoustic signal and a wide temperature range (from 1.6 K to ~700 K). This range is linear from thermal noise to breakdown deformation. To-date, commercial QTFs with a *f_0_* of ~32.76 kHz are used, and only recently the use of custom QTFs in QEPAS based sensor systems were reported [22,23,24]. In this paper, a compact QEPAS sensor using a pigtailed, near infrared DFB diode laser as an excitation source was demonstrated. Two QTFs, with *f_0_* of 30.72 kHz and 38 kHz were investigated and compared to a standard 32.768 kHz QTF. H_2_O was selected as the target analyte. The QEPAS sensor performance using the three different QTFs was experimentally investigated and theoretically analyzed.

## 2. Experimental Setup

**Figure 1:**
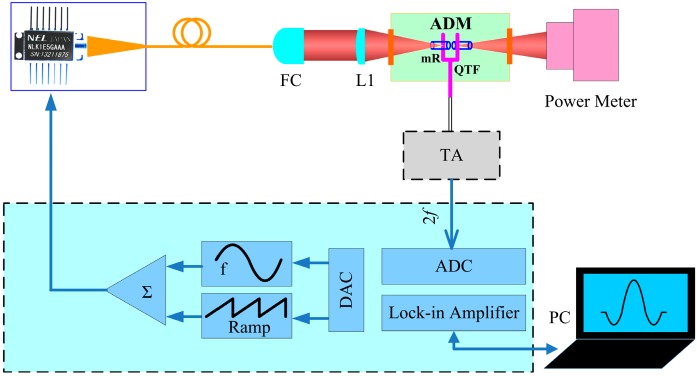
Schematic configuration of a QEPAS-based sensor platform.

A schematic of the QEPAS based sensor platform is shown in Figure 1. A 1.395 μm CW-DFB, fiber-coupled diode laser (NLK1E5GAAA, NEL, Kanagawa-ken, Japan) was employed as the excitation source. The diode laser beam was collimated using a fiber collimator (FC). Subsequently the laser beam was focused between the QTF prongs by using of a plano-convex CaF_2_ lens (L1) with a 40 mm focal length. The remainder of the laser beam was directed to an optical power meter (PS19Q, Coherent) and used for alignment verification of the QEPAS based sensor system. Wavelength modulation spectroscopy (WMS) with 2nd harmonic detection [25] was utilized for sensitive concentration measurements. Modulation of the laser current was performed by applying a sinusoidal dither to the direct current ramp of the diode laser at half of the QTF resonance frequency (*f* = *f_0_*/2). The piezoelectric signal generated by the QTF was detected by a low-noise transimpedance amplifier with a 10 MΩ feedback resistor and converted into a voltage. Subsequently this voltage was transferred to a custom built control electronics unit (CEU). The CEU provides the following three functions: (1) measurement of the QTF parameters, *i.e.*, the quality factor *Q*, dynamic resistance *R*, and resonant frequency *f_0_*; (2) modulation of the laser current at the frequency *f* = *f_0_*/2; and (3) measurement of the 2*f* component generated by the QTF.

**Figure 2:**
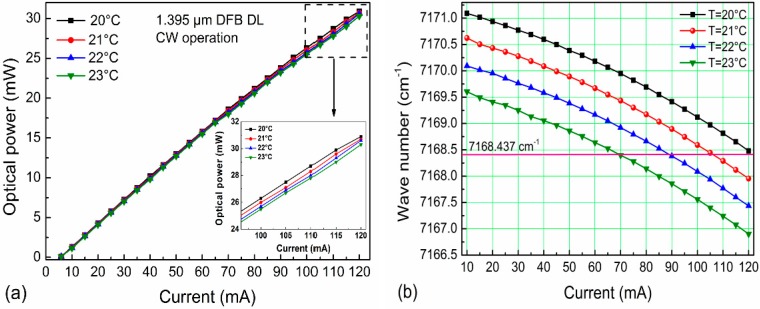
NLK1E5GAAA diode laser output performance at different temperatures: (**a**) Optical power as a function of current; (**b**) Laser current tuning plots.

The optical power emitted by the NLK1E5GAAA diode laser operating with 120 mA drive current was ~30 mW (see Figure 2a were −0.51 cm^−1^/°C and −0.0246 cm^−1^/mA, respectively. The DFB diode laser can be current tuned to target the H_2_O absorption line at 7168.4 cm^−1^ (see Figure 2b), which is free from spectral interference by other molecular trace gas species. The optimum temperature for the highest diode laser power at the absorption line was 21 °C.

## 3. Results and Discussion

**Figure 3:**
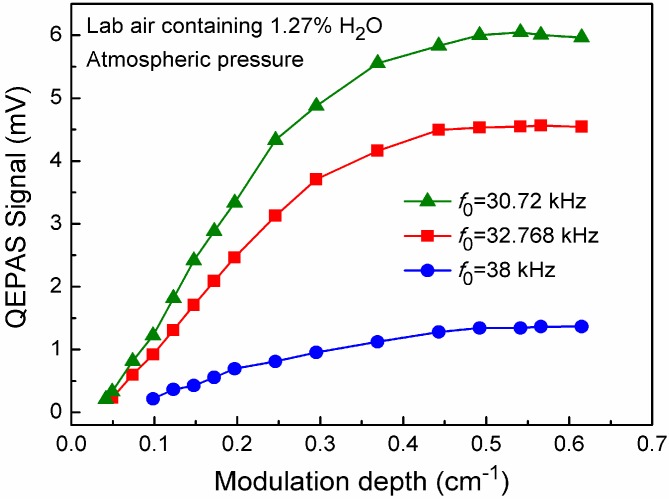
Measured QEPAS signal amplitude as a function of laser modulation depth for three QTFs with different *f*_0_.

The geometries of length, width and thickness of QTF prongs with *f_0_* of 30.72 kHz, 32.768 kHz and 38 kHz, and their respective gap between the two prongs are listed in Table 1. Using these three different QTFs, the performance comparison of QEPAS sensor without the mR architecture was carried out. The integration time for the QEPAS sensor with the three different QTFs was 1 s. Furthermore to obtain maximum 2f QEPAS signals, the laser wavelength modulation depth was optimized. For the purpose of determining the optimum operating conditions of the QEPAS-based sensor system, laboratory air was employed, which contained 1.27% H_2_O as determined by means of a direct absorption method. The experimental results shown in Figure 3 illustrate the influence of the laser modulation depth on the QEPAS signal measured at the targeted 7168.4 cm^−1^ H_2_O absorption line. At the same modulation depth, the QEPAS signal level for a QTF with *f_0_* of 30.72 kHz was larger than that for the other two QTFs with *f_0_* of 32.768 kHz and 38 kHz, respectively. The QEPAS signal amplitude increased with the modulation depth, but when the modulation depth was higher than 0.492 cm^−1^ (20 mA), no further significant change was observed. Therefore, a modulation depth of 0.492 cm^−1^ was found to be optimum.

**Table 1:** Parameters of geometries for three different QTFs.

QTF with *f_0_* (kHz)	Length (mm)	Width (mm)	Thickness (mm)	Gap (mm)
30.72	3.9	0.62	0.36	0.32
32.768	3.6	0.6	0.36	0.3
38	3.5	0.6	0.36	0.34

**Figure 4:**
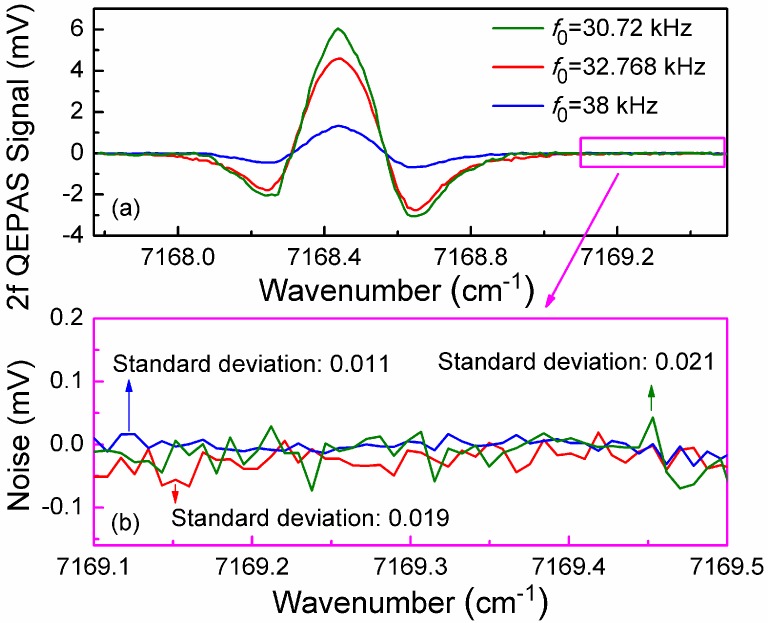
Measured 2*f* QEPAS signal and noise at modulation depth of 0.492 cm^−1^ for three QTFs with different *f*_0_.

The measured 2*f* QEPAS signal and noise at a modulation depth of 0.492 cm^−1^ for three QTFs with different *f_0_* of 30.72 kHz, 32.768 kHz and 38 kHz is shown in Figure 4a,b, respectively. The noise was determined from the signal far from the targeted absorption line. The QEPAS sensor using the 30.72 kHz QTF achieved the maximum signal level. The noise amplitude is different for each of the three QTFs. The signal-to-noise ratios (SNRs) of the three sensors are listed in Table 2. The performance parameters of the QTFs at atmospheric pressure were measured and are also shown in Table 2. From Table 2, it is apparent that the QEPAS sensor employing a 30.72 kHz QTF has a minimum *f_0_* and a maximum *Q* value. Therefore, the effective integration time was maximum, and the QEPAS sensor reached the highest signal level. However, because the 30.72 kHz QTF has a minimum *R*, the noise amplitude was the largest on the basis of Equation (1). The SNR calculated from the measured results were 295, 244, and 127 for QTFs with *f_0_* of 30.72 kHz, 32.768 kHz and 38 kHz, respectively. Based on the data shown in Figure 4 and Table 2, *f_0_* for the QEPAS sensor was lower, the noise level and signal level were higher, but most important of all, the SNR was also higher. Hence QTFs with a smaller *f_0_* can result in improved QEPAS performance. The detection bandwidth Δ*f_0_* for QTF was determined by measuring the QTF resonance curve, and is also listed in Table 2.

**Table 2:** Parameters of three different QTFs at atmospheric pressure.

QTF with *f_0_* (kHz)	Measured *R* (kΩ)	Measured *Q*	Measured Δ *f_0_* (Hz)	Calculated SNR
30.72	89.7	7995	3.75	295
32.768	162.3	6857	4.77	244
38	1469.8	4672	8.13	127

**Figure 5:**
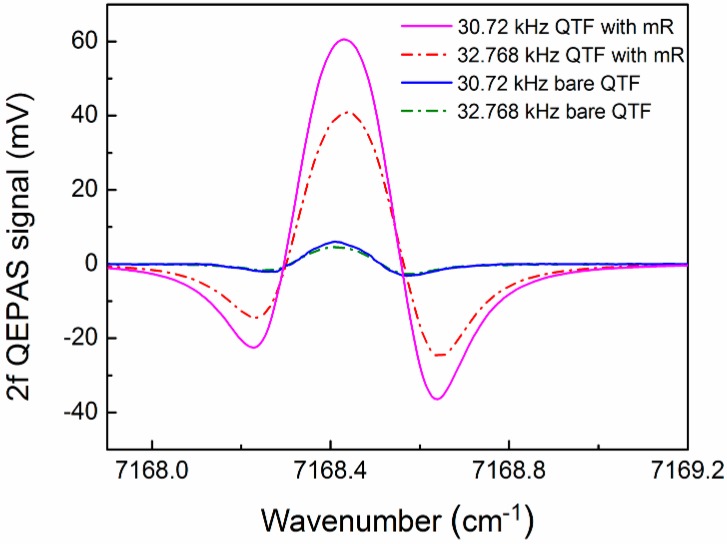
Measured 2*f* QEPAS signals with and without mR at a modulation depth of 0.492 cm^−1^ using QTFs with *f*_0_ of 30.72 kHz and 32.768 kHz.

In the following experiments, a QTF with *f_0_* of 30.72 kHz was utilized for the best sensor architecture. A significant enhancement of the QEPAS signal can be achieved when two metallic tubes acting as a micro-resonator (mR) are added to the QTF sensor architecture [26]. In our experiment, the length and inner diameter of the mR tubes were selected to be 4 mm and 0.5 mm, respectively. Two mR tubes were added to the sides of QTF in order to improve the QEPAS signal. The gaps between the QTF and mR tubes were chosen to be 25 μm. The measured 2*f* QEPAS signals with and without mR at a modulation depth of 0.492 cm^−1^ is shown in Figure 5. In order to make a comparison with the standard QTFs with a *f_0_* of 32.768 kHz, the QEPAS signal measured using a standard QTF equipped with the mR architecture was also depicted in Figure 5. The dimensions of mR tubes used for the QTF with *f_0_* of 32.768 kHz were the same as that for a QTF with *f_0_* of 30.72 kHz. The QEPAS signal had approximately 10-fold and 9-fold enhancement after the addition of the two mR tubes for these two QTFs with *f_0_* of 30.72 kHz and 32.768 kHz, respectively. This resulted in a minimum detection limit of 4.3 ppmv and 5.9 ppmv. The difference between signal enhancements resulted from the effect of different acoustic wave wavelengths on the acoustic resonance in the mR tubes when QTFs with different *f_0_* of 30.72 kHz and 32.768 kHz were used.

## 4. Conclusions

A compact H_2_O QEPAS sensor using a 1.395 μm, pigtailed, CW DFB diode laser was demonstrated as an excitation source. Wavelength modulation spectroscopy and a 2nd harmonic detection technique were used to reduce the sensor background noise. A comparison of three kinds of QTF with different *f_0_* of 30.72 kHz, 32.768 kHz, and 38 kHz as acoustic wave transducers was investigated for the first time. The QEPAS sensor performance was experimentally investigated and theoretically analyzed based on measurements of the QTFs’ parameters. We found that the lower the *f_0_* for a QTF based QEPAS sensor, the higher the SNR becomes. Therefore, QTFs with a smaller *f_0_* can improve the QEPAS sensor performance. However, when *f_0_* is much lower than the standard 32.768 kHz, the effect of environmental acoustic noise on QEPAS sensor noise level become obvious. In this research, a minimum detection limit of 5.9 ppmv and 4.3 ppmv were achieved when QTF with *f_0_* of 32.768 kHz and 30.72 kHz was employed, respectively. The detection limit can be further improved when a QTF with lower *f_0_* and a stronger absorption line is adopted.
